# Autophagy and Ciliogenesis

**DOI:** 10.31662/jmaj.2021-0090

**Published:** 2021-07-06

**Authors:** Yasuhiro Yamamoto, Noboru Mizushima

**Affiliations:** 1 Department of Biochemistry and Molecular Biology, Graduate School of Medicine, The University of Tokyo, Tokyo, Japan; 2 Department of Respiratory Medicine, Graduate School of Medicine, The University of Tokyo, Tokyo, Japan

**Keywords:** autophagy, ciliogenesis, ciliopathy

## Abstract

Autophagy is a major intracellular degradation system and plays important roles in various physiological processes such as metabolic adaptation and intracellular homeostasis. It degrades intracellular components both randomly and selectively. Autophagic activity is tightly regulated primarily by nutrient availability, but also by other extracellular and intracellular signals. Growing evidence suggests that there are multiple links between autophagy and the primary cilium. The primary cilium is an organelle present on the cell surface and is important for keeping cellular integrity by transducing extracellular stimuli inside the cell. Recent studies have revealed that autophagy selectively degrades the ciliogenesis inhibitory proteins OFD1 and MYH9, promoting ciliogenesis. Conversely, autophagy also inhibits ciliogenesis under growth conditions. The primary cilium can also regulate autophagic activity. These findings suggest that the relationship between autophagy and the primary cilia is bidirectional, and that both are important for maintaining the normal function of various organs.

## Macroautophagy is an Intracellular Degradation System

**Figure 1.**
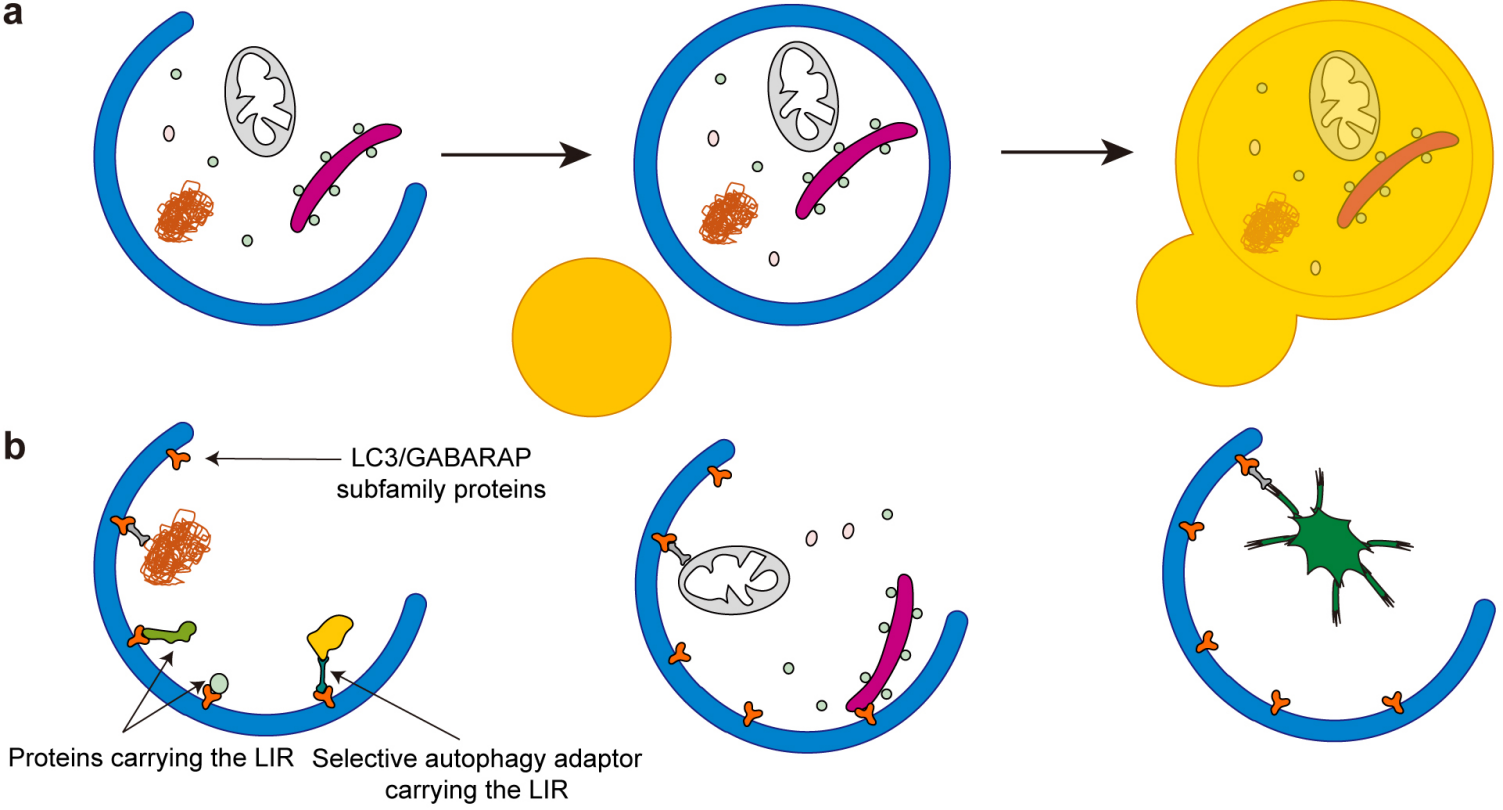
Overview of the autophagy process. a, In the process of autophagy, the isolation membrane expands and engulfs cytoplasmic components. It finally closes to become an autophagosome. The outer autophagosomal membrane fuses with lysosomes to degrade the sequestered materials. b, Various types of selective autophagy. Selective cargo containing LIRs is recognized by LC3 and GABARAP subfamily proteins on the autophagosomal membrane. Alternatively, autophagy adaptor proteins mediate interaction between cargo and autophagosomes. Examples of selective cargo include certain proteins and protein aggregates (left), organelles such as mitochondria and the endoplasmic reticulum (middle), and intracellular bacteria (right).

Macroautophagy (hereinafter, autophagy) is an intracellular degradation process by which cytoplasmic components are degraded in the lysosome (Figure 1a). Autophagy plays key roles in various physiological processes including metabolic adaptation, development, and tissue homeostasis as well as in pathological conditions such as neurodegenerative diseases, muscle and liver diseases, and cancers ^(1), (2)^. When autophagy is induced, contents of the cytoplasm are enclosed by a small membrane sac called the isolation membrane. When the membrane is closed, it becomes a double membrane structure called the autophagosome (Figure 1a) ^(3), (4)^. The autophagosome then fuses with lysosomes to degrade the engulfed material. Autophagy is typically induced by nutrient starvation, which is mainly mediated by the mechanistic target of rapamycin (mTOR), a serine-threonine kinase that positively regulates cell growth and proliferation by promoting anabolic activities, and negatively regulates autophagy by inhibiting upstream autophagy regulators ^(5)^.

Although autophagy was previously viewed as primarily a non-selective intracellular degradation system, recent studies have shown that autophagy also degrades certain substrates selectively (Figure 1b) ^(6), (7)^. Cargo (or substrates) of selective autophagy are soluble and aggregated proteins; organelles (often damaged), including mitochondria, the endoplasmic reticulum, peroxisomes, and lysosomes; and even intracellular pathogens ^(8), (9), (10), (11), (12)^. Most of this cargo is recognized by autophagosomal proteins via ATG8 family proteins, which are classified into the following two subfamilies in mammals: LC3 (including LC3A, LC3B, and LC3C) and GABARAP (including GABARAP, GABARAPL1, and GABARAPL2). These ATG8 family proteins are covalently conjugated to phosphatidylethanolamine in the autophagic membrane and bind to selective cargo that has a motif called the LC3-interacting region (LIR) (Figure 1b) ^(13), (14)^. In some cases, cargo is indirectly recognized by ATG8 proteins through LIR-containing selective autophagy adaptors such as SQSTM1 (also called p62), NBR1, NDP52, optineurin, and TAX1BP1, which interact with both ATG8 and selective autophagy cargo simultaneously ^(6), (7)^. These selective autophagy adaptors are also degraded along with their cargo by autophagy.

## The Primary Cilium is a Cellular Antenna Essential for Cellular Homeostasis

**Figure 2.**
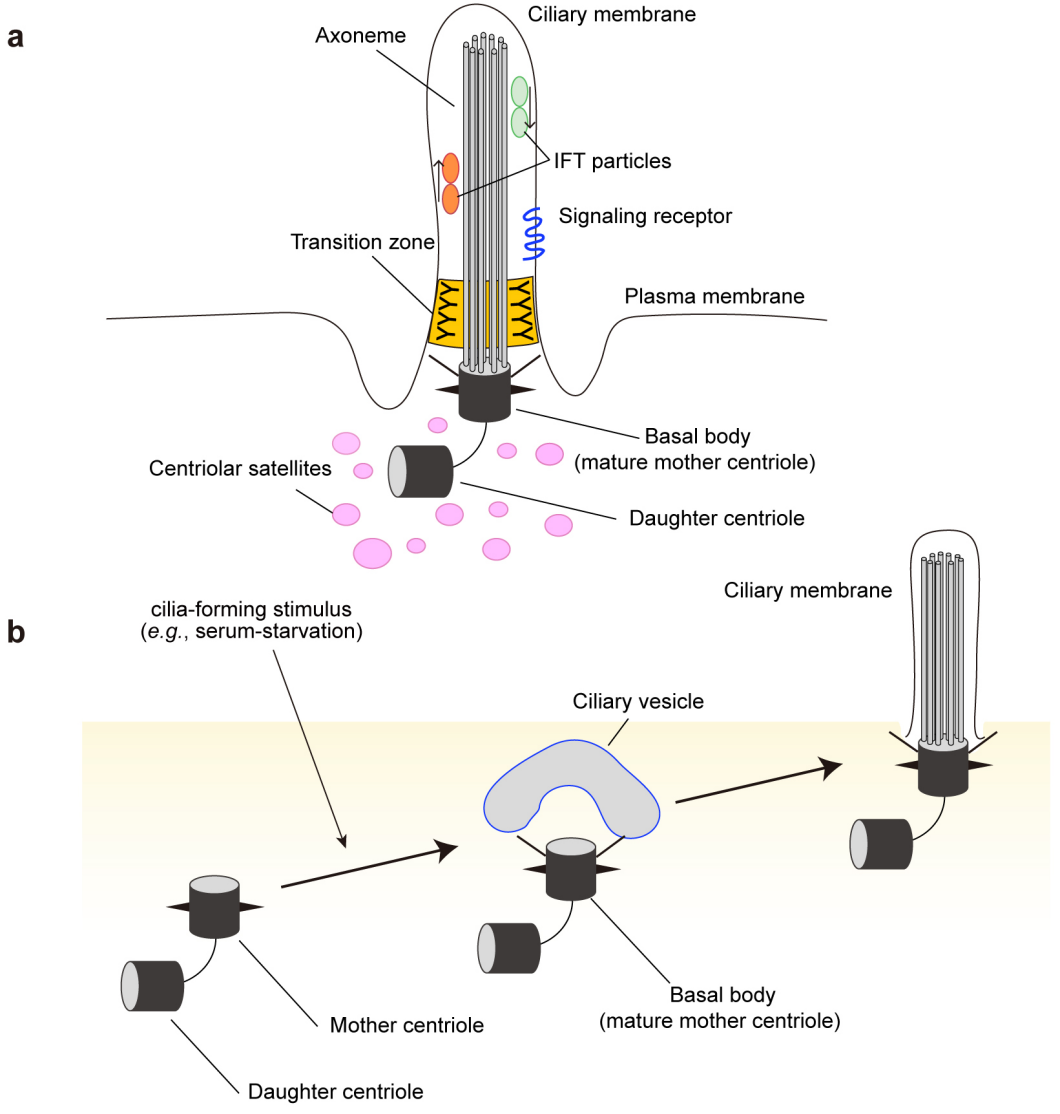
Structure of the primary cilium. a, The primary cilium is composed of a microtubule-based structure called the axoneme, which is ensheathed in an extension of the plasma membrane called the ciliary membrane. The axoneme of the primary cilium is typically composed of nine doublet microtubules. These microtubules extend from the basal body (mature mother centriole). There is a centrosome, consisting of two centrioles, that is surrounded by centriolar satellites, membraneless organelles that localize and move around the centrosome and cilia. The transition zone functions as a barrier that tightly regulates the entry and exit of proteins. Primary cilia are assembled and maintained by intraflagellar transport (IFT) proteins that are transported along the ciliary microtubules by motor proteins. Various signaling receptors are present in the ciliary membrane to respond to extracellular stimuli. b, In response to a cilia-forming stimulus such as serum starvation, cells exit the cell cycle and form primary cilia. The basal body migrates toward the plasma membrane and forms the ciliary vesicle, part of which matures to the ciliary membrane. Finally, the basal body docks to the plasma membrane and the axoneme extends to generate the cilium.

The primary cilium is a hair-like, microtubule-based organelle present on the surface of almost all vertebrate cells ^(15)^. It is composed of a basal body, a matured form of the mother centriole of the centrosome, and a microtubule-based structure called the axoneme (Figure 2). At the base of the cilium, there is a transition zone that serves as a barrier to tightly regulate protein entry to and exit from the ciliary compartments. Inside the cilium, ciliary proteins are transported back and forth by intraflagellar transport (IFT) machinery, including various kinesins and dyneins (Figure 2).

The primary cilium appears to sense, transduce, and integrate a variety of extracellular signals ^(16), (17), (18)^. Extracellular signals conveyed by primary cilia include mechanical stresses (bending of the cilium by fluid flow and tissue deformation), signaling proteins (e.g., Hedgehog [Hh], Wnt, Notch, platelet-derived growth factor, G protein-coupled receptors, growth factors, and hormones). In specialized cells, primary cilia can even respond to specific stimuli, including light, temperature, gravity, and osmolality ^(19)^. The Hh pathway is one of the most characterized cilium-dependent signaling pathways. It plays an essential role in regulating cell fate, stem cell renewal, and carcinogenesis, in addition to development and tissue homeostasis ^(20), (21)^. While the Hh pathway completely depends on primary cilia ^(22)^, other pathways, including the mTOR complex 1 (mTORC1) pathway, function through both cilium-dependent and -independent mechanisms. Signal transmission in the cilia regulates various physiological and developmental processes, such as embryonic patterning, tissue homeostasis, cell differentiation, and organogenesis ^(16), (19), (23)^.

Ciliary dysfunction leads to genetic syndromes known as ciliopathies. They present overlapping phenotypes including renal, hepatic and pancreatic cysts, skeletal defects, retinal degeneration, obesity, hearing loss, intellectual disability, and brain malformations ^(24), (25)^. Examples of ciliopathies include autosomal dominant and autosomal recessive polycystic kidney diseases, Bardet-Biedl syndrome (BBS), oral-facial-digital (OFD) type 1 syndrome, Joubert syndrome, nephronophthisis, Meckel-Grouber syndrome, and Birt-Hogg-Dubé (BHD) syndrome ^(24)^. In addition to these canonical ciliopathies, ciliogenesis appears to be compromised in various human cancers, including melanoma and pancreatic, breast, and renal cell cancers ^(26), (27)^.

Recent studies have shown that primary cilia, ciliary signaling pathways, and cilia-related proteins regulate autophagy and that, conversely, autophagy regulates ciliogenesis ^(28)^. In this review, we discuss recent findings on the crosstalk between cilia and autophagy. The possible involvement of cilia and autophagy in diseases is discussed in more detail in other studies ^(28)^.

## Autophagic Degradation of Certain Proteins Promotes Ciliogenesis

While it has been known for a long time that serum starvation promotes ciliogenesis, the underlying mechanism remains unclear ^(29)^. Recent studies have discovered that autophagy, which is triggered in response to serum starvation, contributes to ciliogenesis by degrading specific proteins.

OFD1 was the first protein reported to be degraded by autophagy to promote ciliogenesis. Mutations in the *OFD1* gene cause OFD syndrome ^(30)^. OFD1 localizes to the centriole and centriolar satellites that surround the centriole and has two opposing functions based on its localization ^(31), (32)^. OFD1 at the centriole acts as a promotor of ciliogenesis by facilitating migration of the basal body to the plasma membrane and allowing the recruitment of essential ciliary components such as IFT88 to the basal body ^(31), (33)^. Conversely, OFD1 at the centriolar satellites suppresses ciliogenesis by inhibiting the recruitment of ciliary proteins essential for cilia elongation such as BBS4. Under cilia-forming serum starvation conditions, autophagy degrades OFD1 at the centriolar satellites and promotes ciliogenesis ^(32)^. When autophagy is blocked, OFD1 accumulates at the centriolar satellites even in serum starvation conditions and impedes the recruitment of proteins required for cilia elongation, leading to impaired ciliogenesis. These results have led to the discovery of autophagy-mediated degradation of a cilia-related protein as a possibly important step in ciliogenesis. A recent study has shown that OFD1 has a LIR to interact with LC3, indicating that OFD1 at centriolar satellites is directly degraded by selective autophagy ^(34)^.

NIMA-related kinase 9 (NEK9) was also identified as a cilia-related protein that is degraded by selective autophagy ^(35)^. NEK9 has a LIR to interact with LC3 and GABARAP family proteins, and associates with autophagic membranes in a LIR-dependent manner ^(35), (36)^. Ciliogenesis was found to be impaired in cells harboring a mutation in the LIR of NEK9. Similarly, primary cilia formation was affected in the kidneys of mice carrying the same mutation. NEK9 interacts with MYH9 (also known as myosin IIA), a negative regulator of ciliogenesis ^(37)^, and mediates autophagic degradation of MYH9; in other words, NEK9 functions as a selective autophagy adaptor for MYH9. MYH9 suppresses ciliogenesis by stabilizing the actin network and inhibiting actin remodeling, which is essential for the early phase of ciliogenesis^(38), (39)^. Thus, autophagic degradation of NEK9-MYH9 is a prerequisite for the proper progression of the early phase of ciliogenesis.

**Figure 3.**
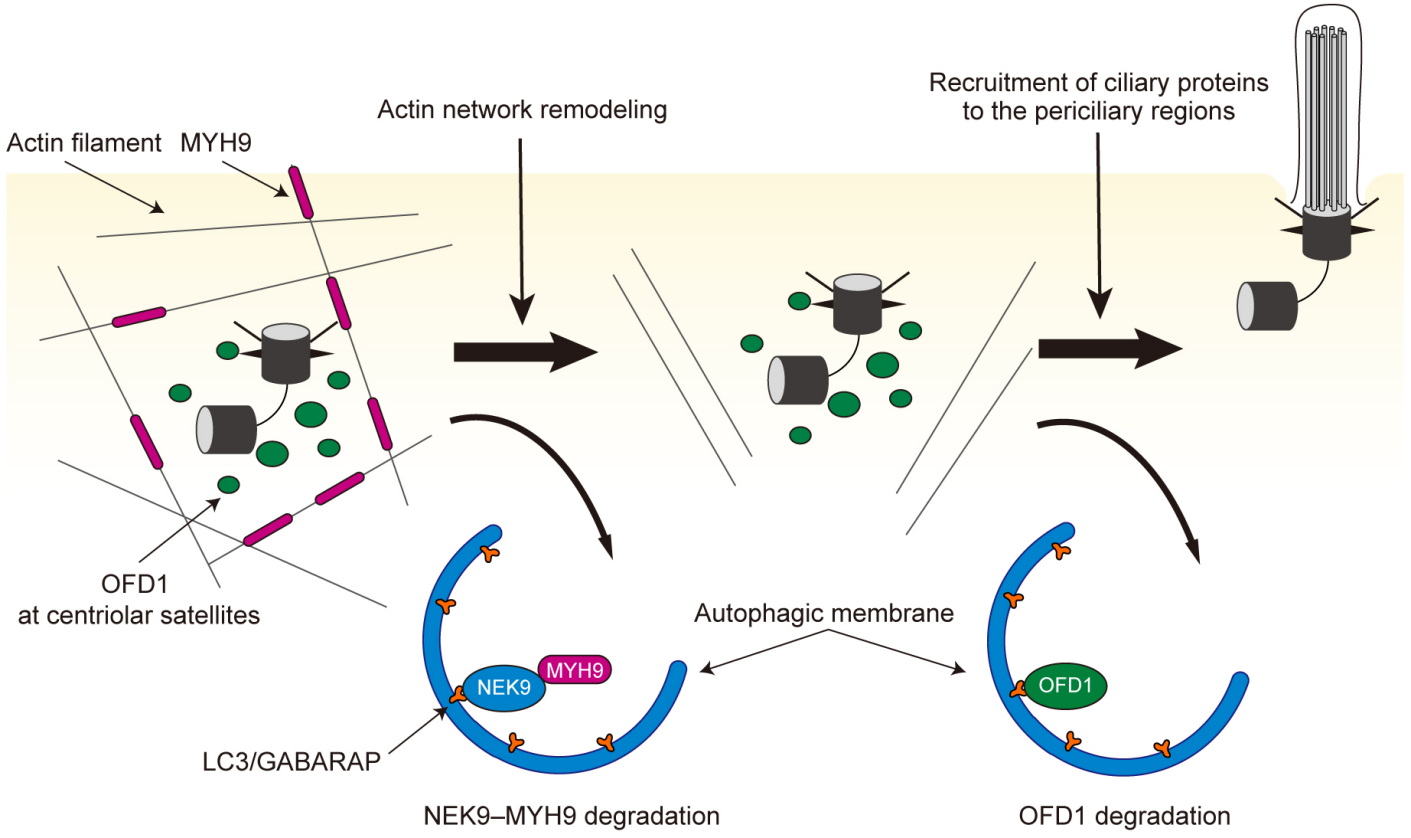
Multi-step regulation of ciliogenesis by autophagy. In growth conditions, MYH9 stabilizes the intracellular actin filament network and inhibits ciliogenesis. Under cilia-forming starvation conditions, NEK9, as a selective autophagy adaptor, mediates autophagic degradation of MYH9. MYH9 degradation promotes actin network remodeling, which is essential in the early phase of ciliogenesis. Independently, OFD1 at centriolar satellites is degraded by autophagy, which allows the recruitment of cilia-related proteins such as BBS4 toward the periciliary regions. Autophagic degradation of both NEK9-MYH9 and OFD1 is required for efficient ciliogenesis.

Autophagic degradation of NEK9-MYH9 and OFD1 is independent; NEK9-MYH9 and OFD1 at centriolar satellites seem to inhibit different steps in ciliogenesis. These findings have revealed that autophagy could play key roles at multiple stages of cilia formation (Figure 3) ^(35)^. Consistent with these findings, upregulation of autophagy promotes ciliogenesis in cells maintained in various culture conditions ^(32), (40), (41), (42), (43)^. It remains to be investigated whether NEK9-MYH9 and OFD1 are modified to be subjected to autophagy upon cilia-forming stimuli. Post-translational modification around the LIR is a well-known mechanism that conditionally regulates the activity of selective autophagy by changing the binding affinity between ATG8 proteins and the LIR ^(7)^. Thus, degradation of these cilia-related proteins by selective autophagy may be similarly regulated by post-translational modifications. Very recently, it was reported that ATG16L1 interacts with IFT20 and regulates ciliogenesis ^(44)^. However, this function appears to be independent of canonical autophagy.

## Basal Autophagy Inhibits Ciliogenesis

Conversely, some reports have demonstrated that autophagy could negatively regulate ciliogenesis ^(45), (46)^. Under normal nutrient-rich conditions, autophagy occurs at basal levels and prevents unwanted cilia formation during cell proliferation by degrading proteins that are essential for cilia formation and assembly ^(45), (47)^. Autophagy-related proteins could localize at cilia or periciliary regions ^(45)^.

These findings suggest that the effects of autophagy on ciliogenesis may vary depending on various experimental conditions in culture cells, and that evaluation by physiologically relevant methods such as using mouse models would be more important than using only culture cells. Because primary cilia formation is impaired in the kidney of autophagy-deficient mice such as *Atg5^−/−^;NSE-Atg5* and *Atg7*^flox/flox^*;PEPCK-Cre* mice, it seems reasonable to assume that, in general, autophagy positively regulates cilia formation under physiological conditions *in vivo* ^(35), (42)^.

## Primary Cilia Regulate Autophagy

**Figure 4.**
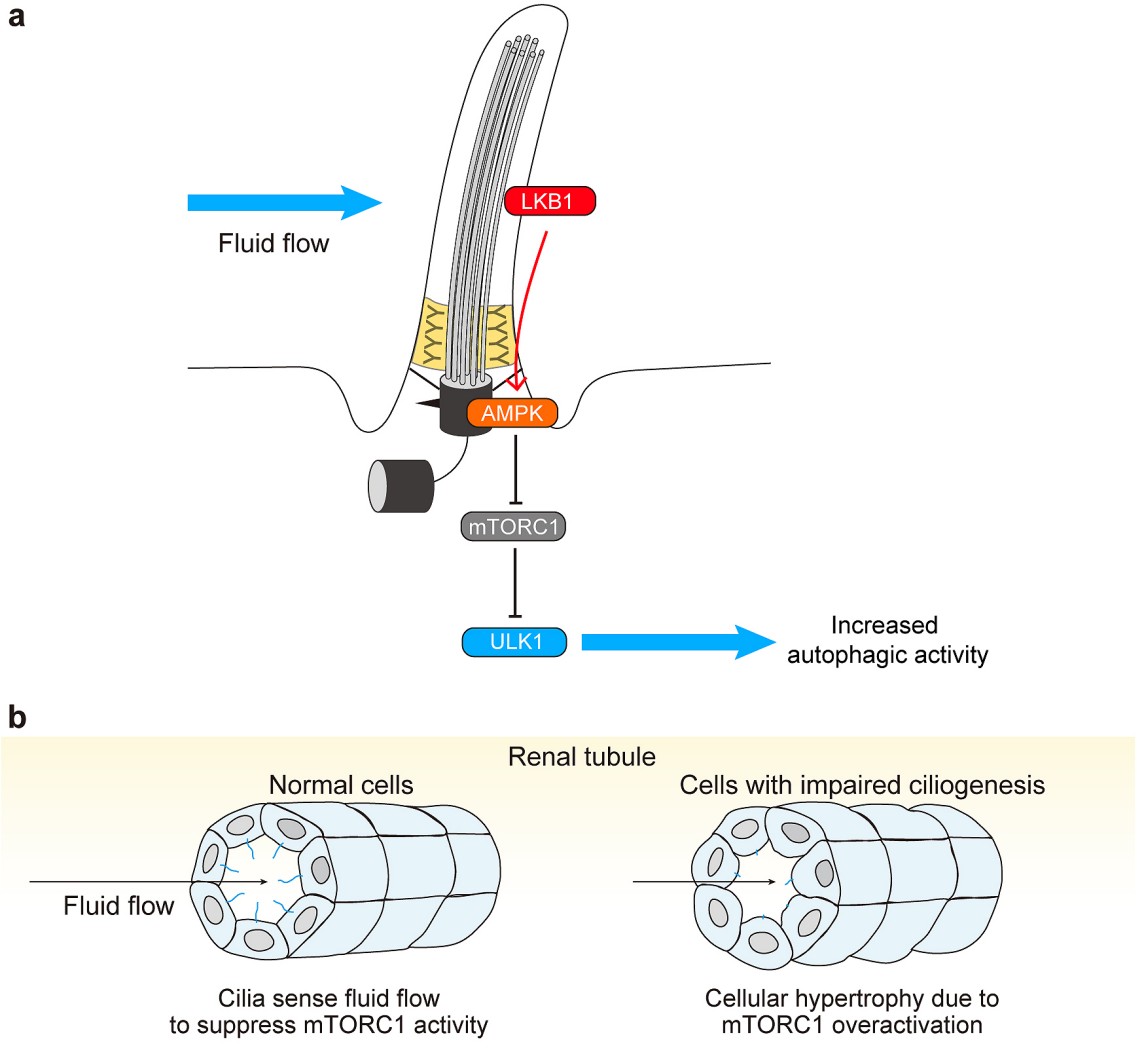
Primary cilia regulate autophagy. a, Primary cilia sense extracellular shear stress induced by fluid flow and activate liver kinase B1 (LKB1) at the ciliary compartment. LKB1 activates AMP-activated protein kinase (AMPK) at the basal body, which inactivates mTORC1 and activates the ULK1 kinase complex, leading to autophagy upregulation. b, Normal renal epithelial tubular cells of renal tubules form primary cilia on the lumen side to sense fluid flow (urine) and suppress mTORC1 activity. Cells with defective primary cilia fail to sense fluid flow and show cellular hypertrophy due to mTORC1 overactivation.

Although autophagy regulates ciliogenesis, the relationship between autophagy and cilia is not unidirectional; primary cilia can also regulate autophagic activity. Because autophagy is activated by suppression of mTORC1 activity, which is regulated by primary cilia, it is plausible that primary cilia regulate autophagy (Figure 4a) ^(48)^. Autophagy activation upon serum starvation requires the presence of functional primary cilia; culture cells with impaired ciliogenesis, for example IFT20- and IFT88-depleted cells, exhibit decreased autophagy upon serum removal ^(45)^.

In normal ciliated cells, such as renal epithelial cells, primary cilia transduce extracellular shear stress induced by fluid flow and activate liver kinase B1 (LKB1), which localizes to the ciliary compartment and the basal body. LKB1 activates AMP-activated protein kinase localized at the basal body, which inactivates mTORC1 and activates the ULK1 kinase complex, leading to autophagy upregulation and cell size reduction (Figure 4a) ^(49), (50)^. Accordingly, cells with defective primary cilia show cellular hypertrophy (Figure 4b) ^(49), (51)^.

****There is emerging evidence suggesting that primary cilium-dependent autophagy governs physiological adaptations in organs. A recent study revealed a key role for primary cilium-dependent autophagy in the eye ^(52)^. Induction of autophagy elicited by high intraocular pressure (IOP) and mechanical stretch of trabecular meshwork cells have been known to be essential in IOP ^(53)^. Shim et al. discovered that primary cilia act as a mechanosensor for mechanical stretch-induced autophagy and identified cross-regulatory talk between AKT1 and noncanonical SMAD2/3 signaling as critical elements of primary cilia-mediated autophagy activation by mechanical stretch ^(52)^. Removal of primary cilia disrupts the homeostatic IOP compensatory response and prevents autophagy induction in response to elevated pressure challenges, which supports the role of primary cilia-mediated autophagy in regulating IOP homeostasis.

In addition, another recent report has shown that shear stress caused by fluid flow stimulates lipophagy (selective degradation of lipid droplets by autophagy) in renal epithelial cells in a cilium-dependent manner ^(54)^. It facilitates the production of fatty acids that provide mitochondrial respiratory substrates through β-oxidation to generate ATP. Primary cilium-dependent lipophagy and mitochondrial biogenesis are required to support energy-consuming cellular processes that include glucose reabsorption, gluconeogenesis, and cytoskeletal remodeling. These findings reveal the involvement of primary cilia and autophagy in the transduction of extracellular mechanical forces into intracellular metabolic adaptation. The initiation of a specific type of autophagy by primary cilia in response to various extracellular stimuli (e.g., fluid flow, light, pressure, temperature, nutritional, and hormonal status) may be important for cells to adapt to different environments during differentiation and development. Indeed, another piece of evidence demonstrates that inhibition of cilia-mediated autophagy blocks proper neuroectodermal differentiation, suggesting a key role for primary cilium-dependent autophagy in differentiation ^(55)^.

## Cilia-related Proteins Regulate Autophagic Activity

Multiple studies have suggested that cilia-related proteins are directly involved in autophagy, not just in ciliogenesis. Inositol 5-phosphatase (*INPP5E*), a causative gene of Joubert syndrome, is a positive regulator of autophagosome maturation^(56), (57)^. Initially, INPP5E was identified as a cilia-related protein that localizes to primary cilia. INPP5E depletion results in shorter cilia and affects transmission of Hh signaling ^(58), (59)^. A recent study has revealed that INPP5E mutations, which impair phosphatase activity, affect autophagic activity by disturbing autophagosome-lysosome fusion, suggesting the direct involvement of cilia-related protein in autophagy ^(57)^. In addition, another cilia-related protein called inositol phosphate-5-phosphatase, known as OCRL, which localizes to basal bodies and cilia and positively regulates ciliogenesis, also plays a functional role in autophagy ^(60), (61)^. *OCRL* is a causative gene for Lowe syndrome and Dent-2 disease, in which fibroblasts present impaired ciliogenesis ^(62)^. Similar to INPP5E, OCRL also regulates autophagosome-lysosome fusion; loss of catalytic activity of OCRL causes autophagosome accumulation and lysosomal dysfunction in cells isolated from patients with Lowe syndrome ^(61)^.

Folliculin (FLCN) localizes to primary cilia and is essential for ciliogenesis and cilium-dependent signaling such as the Wnt and planar cell polarity pathways ^(63)^. Mutations in the *FLCN* gene cause BHD syndrome ^(64)^. In addition to its ciliary function, FLCN also localizes to lysosomes and acts as an activator of RagC/D GTPases ^(65)^. Kidney samples from patients with BHD show autophagic defects ^(66)^. A recent study indicated that FLCN is crucial for mTORC1-mediated phosphorylation of transcription factor EB (TFEB), a master regulator of lysosomal biogenesis and autophagy, and that abnormal constitutive TFEB activation is the main driver of kidney abnormalities (e.g., renal cysts and renal carcinoma) and mTORC1 hyperactivity in a mouse model of BHD syndrome ^(67)^. Whether overactivation of TFEB is also involved in other clinical manifestations of BHD syndrome is a subject of future research, but some of ciliopathy-related symptoms of BHD may be primarily due to abnormal activation of mTOR and suppression of autophagy rather than to impaired ciliogenesis.

## Conclusion

We have described the bidirectional relationship between autophagy and the primary cilium; autophagy regulates ciliogenesis positively or negatively, likely depending on the cellular context, and primary cilia regulate autophagy. Although there has been significant progress during the past decade, there remain further questions. For example, both autophagy and ciliogenesis are stimulated by serum starvation, but it is unclear whether this is merely a coincidence or whether there is a causal relationship. Amino acid starvation induces autophagic degradation of OFD1 and NEK9-MYH9, but not ciliogenesis, indicating that the degradation of these factors is not sufficient for ciliogenesis and signals triggered by serum starvation would be crucial. It would be valuable to determine whether it is important to achieve acute reduction of OFD1 and MYH9 during ciliogenesis or to keep their basal levels low. Also, there is almost nothing known about the relationship between autophagy and motile cilia. Further studies will expand our knowledge of the cilia-autophagy axis, which is vital for cellular and organ homeostasis.

## Article Information

### 

This article is based on the study, which received the Medical Award of The Japan Medical Association in 2020.

### Conflicts of Interest

None

### Sources of Funding

This work was supported by Exploratory Research for Advanced Technology (ERATO) (No. JPMJER1702 to N.M.) from the Japan Science and Technology Agency (JST).

### Author Contributions

Y.Y. and N.M. wrote the manuscript.
